# Social environment and cardiometabolic health outcomes: systematic review and meta-analysis

**DOI:** 10.1093/eurpub/ckac129.560

**Published:** 2022-10-25

**Authors:** TC Abreu, JWJ Beulens, LJ Schoonmade, JD Mackenbach

**Affiliations:** 1 Department of Epidemiology and Data Science, Amsterdam UMC, Amsterdam, Netherlands; 2 University Library, VU, Amsterdam, Netherlands

## Abstract

A number of studies investigated the relationship between the social environment (SE) (i.e., the social relationships and social context in which groups of people live and interact) and lifestyle behaviours. However, to what extent this relation extends to cardiometabolic disease (CMD) outcomes is unknown. This systematic review and meta-analysis summarizes the available evidence. We systematically searched PubMed (Medline), Scopus, and Web of Science from inception to 16 February 2021. Outcomes of were type 2 diabetes mellitus and cardiovascular diseases and determinants were SE factors. We assessed the quality of the studies with Newcastle-Ottawa Scale (NOS). We meta-analysed exposure-outcome combinations when ≥3 associations from high quality papers were available. Results are expressed as OR, 95%CI. From 7,671 records screened, 208 were included. Of these, 92% were conducted in high income countries, 58% were cross-sectional studies, and 20% were of poor quality. Among the 208 studies, 746 relevant associations were investigated. The largest number of associations investigated was on the dimension Economic and Social Disadvantage (ESD; 59%), followed by Social Relationships and Norms (21%) and Discrimination and Segregation (9%). Less evidence was found for the remaining dimensions. Meta-analysis of 14 exposure-outcome combinations indicated that worse SE was associated with increased odds of CMD outcomes. Despite this tendency, only the association between ESD and heart failure was statistically significant (1.58, 1.11-2.27; n = 4; I2=92%). Generally, heterogeneity was high. In conclusion, higher levels of ESD seem to contribute to increased risk of heart failure. The existing literature is highly heterogeneous and varies notably in terminology. Moreover, the dimensions Social Cohesion and Social Capital, Crime and Safety, Civic Participation and Engagement and Disorder and Incivilities are underexplored in relation to CMD. (PROSPERO-ID: CRD42021223035).

**Key messages:**

• Worse SE was associated with increased odds of CMD outcomes, with higher levels of Economic and Social Disadvantage being statistically significantly associated with increased risk of heart failure.

• The existing literature is highly heterogeneous and varies notably in study design and terminology.

